# Medication adherence among patients with Type 2 diabetes: A mixed methods study

**DOI:** 10.1371/journal.pone.0207583

**Published:** 2018-12-11

**Authors:** Nouf M. Aloudah, Neil W. Scott, Hisham S. Aljadhey, Vera Araujo-Soares, Khalid A. Alrubeaan, Margaret C. Watson

**Affiliations:** 1 Clinical Pharmacy Department, King Saud University, Riyadh, Saudi Arabia; 2 Medical Statistics Team, University of Aberdeen, Aberdeen, United Kingdom; 3 Institute of Health & Society, Newcastle University, Newcastle, United Kingdom; 4 University Diabetes Centre, King Saud University, Riyadh, Saudi Arabia; 5 School of Pharmacy and Pharmacology, University of Bath, Bath, United Kingdom; Public Library of Science, UNITED KINGDOM

## Abstract

**Objective:**

Oral hypoglycemic agents (OHAs) are highly effective in managing Type 2 diabetes if taken appropriately. This study assessed adherence to OHAs among patients with Type 2 diabetes and explored factors associated with adherence behaviour.

**Research design and methods:**

Mixed methods were used comprising a cross-sectional study using the Arabic version of the Morisky Medication Adherence Scale followed by semi-structured interviews using the Theoretical Domain Framework to explore key determinants of adherence.

**Results:**

The cross-sectional study included 395 patients of whom 40% achieved a high level of OHA adherence. Lower adherence was associated with younger age (Odds Ratio (OR) 1.084; 95% CI 1.056 to 1.112), higher numbers of non-OHAs (OR 0.848; 95% CI 0.728 to 0.986) and higher HbA1c levels (OR 0.808; 95% CI 0.691 to 0.943). Semi structured interviews based on the Theoretical Domain Framework were completed with 20 patients and identified a wide range of factors potentially associated with OHA adherence, particularly behavioural related factors (e.g. scheduling medication intake, ability to develop a habitual behaviour), social influences (e.g. acting as a role model, the effect of family support), and gaps in knowledge about diabetes and its management with OHAs.

**Conclusions:**

This unique mixed-methods study has highlighted possible reasons for the low levels of OHA adherence in this patient population. Whilst the theoretically-derived determinants of behaviour illustrate the complexities associated with OHA adherence, they also provide a robust underpinning for future intervention(s) development to improve adherence and maximise patient health outcomes.

## Introduction

Oral hypoglycemic agents (OHAs) are highly effective in managing Type 2 diabetes if taken appropriately [1]. Adherence is defined by the World Health Organisation (WHO) as “the extent to which a person's behaviour–taking medication, following diet, and/or executing lifestyle changes, corresponds with agreed recommendations from a health care provider” [1]. Adherence to diabetes medications is an important factor in achieving good diabetes control and preventing mortality and morbidity [2]. It has been reported that adherence to OHAs varies between 36% and 93% among different populations [3]. There are various methods to assess medication adherence, of which, the Morisky Medication Adherence Scale (MMAS-8) is a widely used self-administered validated tool [4].

Medication adherence is a complex process and is affected by diverse and multiple factors [2]. A meta-analysis of 569 studies identified more than 200 variables associated with medication adherence behaviour [2]. Therefore using a comprehensive analytical approach to better understand the particular barriers to and facilitators of adherence to therapy, is urgently needed.

Saudi Arabia is facing an unprecedented increase in Type 2 diabetes and has one of the highest prevalence rates i.e. 17.6%, in the world with high rates of chronic complications [5]. Although several studies have reported poor glycemic control as a significant factor behind high complication rates, no studies have explored the low OHA adherence is a contributing factor for such poor control in this national population [6]. Therefore, the Medication Adherence in Saudi Arabia (MASA) Study is the first study in Saudi Arabia to assess adherence to OHAs among participants with Type 2 diabetes who are not using insulin and to use a comprehensive framework of theoretical underpinning to explore the factors associated with adherence behaviour.

## Research design and methods

A sequential, explanatory mixed methods approach was used comprising a cross-sectional study followed by theoretically-underpinned semi-structured interviews.

### Cross-sectional study

Participants were recruited from the University Diabetes Centre (UDC), King Saud University, Riyadh, Saudi Arabia. The UDC provides a five-day educational programme to their patients with diabetes and closely monitors selected patients on a daily basis through a home blood glucose monitoring programme (HBGM). Eligible participants were patients aged ≥18 years with confirmed Type 2 diabetes for the past 12 months, who were receiving at least one OHA for 12 months prior to recruitment. Pregnant women, insulin users, participants with hearing or cognitive impairment and non-Arabic speakers were excluded. A nurse screened participants by applying the selection criteria to each individual’s medical record when attending their appointment at the general diabetic clinic of the UDC. If eligible, the participants were invited to participate and directed to talk to the researcher (NMA). The researcher then re-checked the eligibility and provided further information about the study, including the information sheet. participants provided signed consent if they agreed to participate. The Arabic version of the MMAS-8 was used to calculate OHA adherence [4]. The Arabic version had some grammatical errors and corrections were undertaken. The corrected Arabic MMAS-8 version was sent to Professor Donald Morisky to ensure the integrity of the Arabic MMAS-8 version for the comparison of future results and he agreed to its use.

Data were collected through a chart review using a pre-designed data collection form that consisted of two parts. Part 1 included socio-demographic characteristics: age; gender; marital status; income; and education. This part also included the diabetes history, all medications being used and the individual’s Body Mass Index (BMI). The Medication Regimen Complexity Score (MRCI) [6] was calculated for each participant as it was suggested as one of the factors that affect medication adherence. Part 2 of the data collection form included the self-administered MMAS-8. The data collection tool and process was piloted with 32 participants recruited from the UDC clinic. The data from the participants of the pilot study were not used in the final analysis.

The sample size for the MASA-8 study was derived from the results of an earlier study of 1367 participants in the United States [4] which reported high adherence (MMAS = 8) for 15.9% of participants. Assuming a high adherence rate of 15.9%, 400 participants would be needed to estimate this proportion with an accuracy of ± 3.6%, i.e. with a 95% confidence interval of 12.3% to 19.5%.

### Semi-structured interview study

The interview study was conducted to explore barriers to and facilitators of OHA adherence. Purposive sampling was used to derive a maximum variation sample of individuals with different levels of adherence, as well as achieving a representative gender-age mix.

The topic guide was developed using the Theoretical Domain Framework (TDF) which comprises 14 domains proven to be relevant to behaviour and behaviour enactment i.e. any type of behaviour, not solely medication adherence [7,8]. Generic questions proposed to target each of the TDF domains were modified to reflect the topic of interest; participants with Type 2 diabetes and medication adherence in Saudi Arabia. The topic guide was initially developed in English (S1) and later translated into Arabic. It was reviewed by two bilingual professionals (Arabic/English) and piloted with two Saudi participants with Type 2 diabetes in order to assess its clarity and consistency. The interviews were conducted face-to-face, audio-recorded and transcribed verbatim. participants were interviewed until data saturation was achieved and no new codes emerged. Ethical approval was given by the Institutional Review Board (IRB) of the Medical College of King Saud University (E-13-1058) where the participants were recruited, and the College Ethical Review Board (CERB), University of Aberdeen (CERB/2014/1/975). The Strengthening the Reporting of Observational Studies in Epidemiology Statement (STROBE) [9] for reporting cross-sectional studies and the Consolidated Criteria for Reporting Qualitative Research (COREQ) checklists [10] were followed in the reporting of this study (S3 Table).

### Analysis

Data from the cross-sectional study were analysed using Statistical Package for the Social Sciences (SPSS) version 22. Descriptive statistics were used to summarise participants’ demographics and MMAS-8 scores. A MMAS-8 score of 8 was defined as high adherence, 6≤ MMAS <8 as moderate adherence, and a score <6 as low adherence. Ordinal logistic regression was used to predict the association of OHA adherence category with socio-demographic and clinical factors.

Thematic content analysis was undertaken during which the interview transcripts were analysed systematically. First, two interviews were undertaken, transcribed into Arabic and translated to English. The two interviews were analysed independently by authors NMA and VA-S to code for barriers and facilitators to OHA adherence. Next, a further five interviews were transcribed in Arabic and analysed by NMA using the draft coding manual developed from the first two interviews. Additional codes emerged and were linked to the appropriate TDF domains. All quotations selected, and related codes, were translated to English and discussed with VA-S.

The final coding manual with definitions was agreed upon and used for all subsequent interviews. NMA analysed the remaining interviews. Authors NMA and HSA discussed the quotations in Arabic and their related codes and selected the quotations presented.

All the above steps were conducted independently by a minimum of two researchers to maximize validity. Disagreements were resolved by discussion. NVivo 10 for Windows was used to support the analysis of the first two interviews. NVivo 10 has, however, some limitations as it does not support the Arabic language and therefore could not be used to analyse the remaining interviews. ATLAS.ti 7 worked well and presented fewer problems than NVivo 10. ATLAS.ti 7 was therefore used to support the analysis of the remaining interviews.

## Results

In total, 395 patients participated in the cross-sectional study which was conducted between May and October 2014. The mean (SD) age was 57.8 (8.7) years, the majority were male and were married. The participant’s mean (SD) duration of diabetes was 12.9 (8.0) years and their mean (SD) HbA1c was 7.9% (1.4) or 63 (15.3) mmol/mol. The mean (SD) BMI was 30.5 (6.0) kg/m2.

The mean (SD) number of OHAs was 1.7 (0.7) and the mean (SD) duration of OHA use was 11.8 (7.4) years. More than 40% of the studied cohort were college graduates or higher and more than 30% had a monthly income of less than 10,000 Saudi Riyals (SR) ($2666), (1SR = 0.27 USD) (Table 1).

**Table 1:** Characteristics of participants in the cross sectional study n = 395.

Characteristics Category	Subcategory		MMAS-8 adherence score		
		Total	High = 8	Moderate <8 to 6	Low <6
Number (%)		395 (100)	158 (40.0)	145 (36.7)	92 (23.3)
Age (years) mean (SD)		57.8 (10.7)	62.0 (9.7)	56.9 (9.2)	52.0 (11.4)
BMI (kg/m^2^) mean (SD)		30.5 (6.0)	30.1 (6.2)	31.1 (6.1)	30.3 (5.5)
Duration of diabetes mean (SD)		12.9 (8.0)	14.7 (8.4)	12.4 (7.8)	10.7 (6.9)
Number of OHM taken mean (SD)		1.7 (0.7)	1.7 (0.7)	1.8 (0.7)	1.7 (0.6)
Duration of OHM use (years) mean (SD)		11.8 (7.4)	13.6 (7.7)	11.4 (7.1)	9.5 (6.5)
Number of other medication used mean (SD)		4.1 (2.3)	4.2 (1.9)	4.2 (2.6)	3.7 (2.4)
HbA1c mean (SD)		7.9 (1.4)	7.8 (1.3)	7.9 (1.2)	8.3 (1.7)
MRCI mean (SD)		11.0 (4.0)	11.1 (3.5)	11.1 (4.3)	10.6 (4.4)
Five-day educational programme (%)		203 (51.4)	79 (50.0)	70 (48.3)	54 (58.7)
Enrolled with HBGM (%)	No	269 (68.1)	110 (69.7)	101 (69.7)	58 (63.0)
	Yes, before	91 (23.0)	36 (22.8)	30 (20.7)	25 (27.2)
	Yes, now	35 (8.9)	36 (22.8)	30 (20.7)	25 (27.2)
Gender	Men (%)	236 (59.7)	105 (44.5)	78 (33.1)	53 (22.4)
	Women (%)	159 (40.3)	53 (33.3)	67 (42.1)	39 (22.6)
Education (%)	No	71 (18.0)	33 (20.9)	23 (15.9)	15 (16.3)
	High school or lower	149 (37.7)	56 (35.4)	61 (42.1)	32 (34.8)
	College or higher	175 (44.3)	69 (43.7)	61 (42.1)	45 (48.9)
Marital status (%)	Married	344 (87.1)	137 (86.7)	128 (88.3)	79 (85.9)
	Single*	51 (12.9)	21 (41.2)	17 (33.3)	13 (25.5)
Income (SR) (%)	<5000	53 (13.4)	20 (13.4)	19 (13.6)	14 (15.4)
	>5000–10000	74 (18.7)	37 (24.8)	22 (15.7)	15 (16.5)
	10000–20000	153 (38.7)	58 (38.9)	62 (44.3)	33 (36.3)
	20000–35000	60 (15.2)	16 (10.7)	27 (19.3)	17 (18.7)
	>35000	40 (10.1)	18 (12.1)	10 (7.1)	12 (13.2)

BMI, body mass index; CI, confidence interval; HbA1c, glycosylated haemoglobin; HBGM, home blood glucose monitoring; MMAS, Morisky Medication Adherence Score; MRCI, Medication Regimen Complexity Index (higher score represent higher complexity); OHM, Oral Hypoglycaemic Medication; SD, standard deviation; SR, Saudi Riyals (1 SR = $0.27)

* includes unmarried, divorced and widow.

High (MMAS = 8) levels of OHA adherence were reported in 40% (n = 158), moderate (6≤ MMAS <8) levels with 37% (n = 145) and low (MMAS <6) adherence with 23% (n = 92).

### Factors associated with OHA adherence

Higher levels of adherence were observed with increasing age (OR, 1.084; 95% CI, 1.056–1.112), taking fewer non-diabetic medications (OR, 0.848; 95% CI, 0.728–0.986), and lower HbA1c (OR, 0.808; 95% CI, 0.691–0.943) (Table 2). There was no evidence that the other parameters tested (gender, marital status, education level, income and diabetes duration) were associated with OHA adherence.

**Table 2:** Factors predicting OHM adherence using ordinal logistic regression analysis.

Category	Subcategory	OR (95% CI)	P value
Age (years)		1.084 (1.056–1.112)	**<0.001**
Gender	Women	1	-
	Men	0.753 (0.467–1.215)	0.060
Marital status	Married	1	-
	Single	1.128 (0.539–2.361)	0.750
Education	No	1	-
	High school or lower	0.485 (0.224–1.050)	0.067
	College or higher	0.841 (0.506–1.396)	0.503
Income (SR)	≤5000	1	-
	>5000–10000	2.241 (0.850–5.907)	0.103
	>10000–20000	2.139 (0.938–4.879)	0.071
	>20000–35000	1.404 (0.697–2.825)	0.342
	>35000	0.833 (0.379–1.830)	0.649
BMI (kg/m^2^) N = 394*		1.036 (0.999–1.074)	0.060
Duration of diabetes (years)		1.022 (0.953–1.096)	0.539
HbA1c (%)		0.808 (0.691–0.943)	**0.007**
Number of OHM taken		0.818 (0.564–1.187)	0.290
Duration of OHM use (years)		1.019 (0.944–1.100)	0.632
Number of other medication used		0.848 (0.728–0.986)	**0.032**
Duration between HbA1c test and completion of MMAS-8 (days)		1.001 (0.991–1.011)	0.800
MRCI		1.017 (0934–1.108)	0.698
Completed the five-day educational programme	No	1	-
	Yes	1.089 (0.716–1.657)	0.691
Enrolled with HBGM	No	1	-
	Yes, before	0.916 (0.444–1.892)	0.813
	Yes, now	0.916 (0.413–2.035)	0.830

The bold values represent statistically significant differences (*P<0*.05).

BMI, body mass index; CI, confidence interval; HbA1c, glycosylated haemoglobin; HBGM; home blood glucose monitoring; MMAS, Morisky Medication Adherence Score; MRCI, medication regimen complexity index (higher score represent higher complexity); OHM, Oral Hypoglycaemic Medication; SR, Saudi Riyals (1 SR = $0.27). *missing BMI information for one participant.

### Key factors associated with adherence to OHAs

Half of the participants included in the cross-sectional study (n = 199) were invited to participate in an interview and 54% (n = 108) of them agreed to be interviewed and provided the researcher with their contact details. A list was created that contained the study ID, name, gender, age, adherence level and contact information of the participants who agreed to be interviewed. Then, they were sampled purposively according to the required characteristics following the sequence of the list. Interviews were completed with 20 individuals. Patients were interviewed until data saturation was achieved and no new codes emerged. Data saturation was reached with 17 interviewees and three additional interviews were conducted for assurance. The mean age and range of the included sample was 51.4 (range 26–72) years and 55% of the sample were men with a mean diabetes duration at 11.7 (range 3–20) years, and mean HbA1c of 8% (range 6.9–13.3) or 64 (range 78.1–47.5) mmol/mol (S2 Table).

Thematic content analysis was performed, which involved systematically analysing transcripts, assigning codes to particular ideas (data) and gathering together examples of those codes from the text. Several cycles of coding were needed to reach the final version of the coding manual. For example, the number of codes at the first stage of the analysis was 104, but decreased to 65 and then to 36 in subsequent stages. The codes that emerged were linked back to the domains of the TDF for helping in future mapping of intervention(s). For example, if a patient was described lack of knowledge of the side effects of his/her diabetic medication, this was coded as "knowledge of OHM" and linked to the TDF "knowledge" domain. Similarly, if the patient talked about the role of his/her spouse in reminding him/her to take his medication this was coded as "family support" and linked to the "social influence" domain. The interview results revealed various factors associated with OHA adherence which were clustered around 13 of the 14 TDF domains. The only domain that was not coded on this sample was the “Goals” domain. Table 3 presents the factors identified and example quotations linked to the TDF domains. Each quotation is referenced with the participants study ID, age, gender, adherence and HbA1c levels. Six domains were of particular relevance: behavioural regulation, social influence, knowledge, environmental context, beliefs about diabetes consequences and memory attention and these are presented in more detail below.

**Table 3:** Domains from the TDF and example quotes for barriers and facilitators of adherence behaviour.

TDF Domain	Facilitator of adherence	Barrier to adherence
Behavioural Regulation	Scheduling medication intake “Yes, I rarely forget it; I have one box divided into two places. I’m carrying inside it my diabetes and blood pressure pills.” MASA 143, M, 37 years, MMAS 3.5, HbA1c 6.9%. Being able to develop a habitual/automatic behaviour “In the first 1 to 3 years it’s a big deal, but after 15 years it became a habit, just like smoking; if I eat and don’t take it I think, “Where is the medication? Bring me the medication” ID 125, M, 61 years MMAS 7, HbA1c 8.4%.	Experience of managing hypoglycaemia “I have my own device and the hospital gave me a device and chips so I kept checking the diabetes. I noticed that everything is fine, and that is it [the reason to discontinue the OHM]” ID 143, M, 37 years, MMAS 3.5, HbA1c 6.9%.
Social Influences	The effect of family support “…. if I reject the medications and don’t want to take them, the doctor would speak to my husband or my brother and say take care of her, take her to a shrink, maybe there is some pressure at home, maybe there are problems….” MASA 186, F, 41 years, MMAS 2.5, HbA1c 7.5%. Sharing medication (in case of forgetfulness and/or before collecting a prescription) “I once attended an event and the host [a friend] prepared a plate full of Glucophage [OHM] and said if anyone forgot to take it, here it is on the plate, out of hospitality.” ID 019, M, 49 years, MMAS 8, HbA1c 8%. Having a supportive physician “I try to memorise the medication name so when I talk to the doctor he understands me …, and this gives him more confidence; when he has confidence in me I have confidence in him too … and that is a connection between us”MASA 125, M, 61 years, MMAS 7, HbA1c 8.4%. Acting as a role model “At gatherings I say bring me my medicine to take away shame from the others…if those who hold college degree and also say bring me my medicine…” ID 166, M, 55 years, MMAS 8, HbA1c 7.5%.	Family gatherings and holidays “I have to attend family gatherings, which creates pressure on me. I don’t like when people start asking me what medication I’m taking or what’s wrong with me.” ID 186, F, 41 years, MMAS 2.5, HbA1c 7.5% Physician–patient relationships “…, the first doctor wasn’t good.. you tell him that you feel dizzy, and he says don’t stop the medication. I didn’t go to him again and I keep monitoring myself. I decided that I will never go again…..”. ID 78, M, 26 yeas, MMAS 2.5, HbA1c 7%.
Knowledge	Observing a family member's suffering from diabetic complications because of not adhering to the diabetic medication “Why should I commit [to take OHM], because I lived through a tragedy. …. He [his father] got diabetic gangrene which required that one of his limbs be cut off. This horrified us, and later I discovered by chance that I have diabetes” ID 019, M, 49 years, MMAS 8, HbA1c 8%.	Provide knowledge on diabetes to their own families “If there is something to raise awareness, if there is supportive medication, at least some brochures to educate my wife [about sexual health]….” MASA 019, M, 49 years, MMAS 8, HbA1c 8%.
		Education about OHM, particularly on the medication’s mechanism of action, side effects, and interactions with other drugs “When you take a medicine and you have no idea what it does.. you just take it, …. but when someone tells you all this [about OHM] you become able to notice any change in your body and how to handle it.. you know when routines or food changes..….” MASA 125, M, 61 years, MMAS 7, HbA1c 8.4%.
		Education about medication adherence issues “The UDC gave us two lectures about the medications, they taught us what is the name of the medication and other related information …They should give us tips to motivate ourselves to take the medication not only the information about the medication” MASA 110, M, 33 years, MMAS 3.75, HbA1c 7.7%.
		Knowledge about managing OHM when changes in routines occur “… yesterday I took my medication but I didn’t have an appetite for dinner… so I ate a cheesecake, … the pharmacy gave you the medication and they do not know you, you have to know yourself …..” MASA 110, M, 33 years, MMAS 3.75, HbA1c 7.7%.
Environmental context and resources	Having trust in the UDC and having appropriate follow-up “(…). first of all, after the visits there should be connection through the phone on a weekly basis to check on the patient’s condition, just to make sure that the patient doesn’t stop taking his medications” MASA 186, F, 41 years, MMAS 2.5, HbA1c 7.5%. Being able to have access to medication without a prescription “… I take enough depending on the period I am travelling. If it runs out I get it from the pharmacy, as I already know the name of the medicine. .” ID 125, M, 61 years, MMAS 7, HbA1c 8.4%. Impact of Saudi culture such as religious beliefs, activities, and dedication on OHM adherence. “I take my medicine because it’s a way to worship Allah, because he says don’t throw yourselves in Tahluka [anything that would lead to your death].” ID 166, M, 55 years, MMAS 8, HbA1c 7.5%.	Stress “…I do not want anything to restrict me. If I wasn’t afraid of the diabetes worsening I would stop the regulator [Glucophage, an OHM] also [she stopped all her medication except OHM].” MASA 186, F, 41 years, MMAS 2.5, HbA1c 7.5%.
		Having to manage the multiple intake of several medications “I would like to take one pill instead of two; it has the same medical effect but it makes me feel better”MASA 110, M, 33 years, MMAS 3.75, HbA1c 7.7%.
		The pharmacy service at the UDC. “… This is one of the obstacles; sometimes I need to travel for a conference or a meeting and my medications are about to run out but I still didn’t finish my three months. They would say we can give it to you five days earlier, but no more than this..!” MASA 006, M, 44 years, MMAS 7, HbA1c 7.4%.
		Experiencing a hypoglycaemic incident. “… there was an accident .. A person took the wrong side of the road… we came face to face; I avoided him but the driver on my right couldn’t and they crashed face to face and he died immediately. … they said that .. he had a diabetic attack. It would be a disaster if I have one while driving.” MASA 143, M, 37 years, MMAS 3.5, HbA1c 6.9%.
Beliefs about consequences	Avoiding diabetic complications “.. I’m afraid the complications can affect the kidneys and eyes. …I’m terrified; I would like to ask how I can prevent it from affecting my legs and kidneys.” MASA 168, F, 44 years, MMAS 6, HbA1c 8.8%.	Side effects of OHM ”… I suffer from diabetes and obesity, and these medications contradict what I do, so they did not do me any good and I stopped taking them.” MASA 125, M, 61 years, MMAS 7, HbA1c 8.4%. Not feeling any symptoms of diabetes “I didn’t take it [the OHM] for six months and didn’t feel anything, and when I took it I didn’t feel anything. When I took it I never felt anything, and even when I didn’t take it I felt the same” ID 196, M, 63 years, MMAS 3, HbA1c 13.3%.
	Feeling improved quality of life “No, Alhamdulillah (thank God) I became better; I felt some muscle ache but now it is gone since I started taking the diabetes pills” MASA 184, F, 64 years, MMAS 7, HbA1c 8.7%. Avoiding being prescribed insulin injections “It was a turning point in my life when the doctor said that if the pills don’t work we will have to initiate injections… it made me think I don’t want to take injections for the rest of my life. You can punish me anyway but don’t give me injections.” ID 402, F, 58 years, MMAS 8, HbA1c 7%.	
Memory, attention, and decision processes	A decision to adhere to medication with improved quality of life. “No, Alhamdulillah (thank God) I became better; I felt some muscle ache but now it is gone since I started taking the diabetes pills” MASA 184, F, 64 years, MMAS 7, HbA1c 8.7%.	Forgetfulness “What can I do? I overslept. I don’t want to have dinner and I forgot my dose. This happens to me once or twice a month.” ID 168, F, 44 years, MMAS 6, HbA1c 8.8%. A decision of non-adherence was mentioned in association with different domains such as stress, hypoglycaemic side effect “…I do not want anything to restrict me. If I wasn’t afraid of the diabetes worsening I would stop the regulator [Glucophage, an OHM] also [she stopped all her medication except OHM].” MASA 186, F, 41 years, MMAS 2.5, HbA1c 7.5%. ”… I suffer from diabetes and obesity, and these medications contradict what I do, so they did not do me any good and I stopped taking them.” ID 125, M, 61 years, MMAS 7, HbA1c 8.4%.
Optimism	Awareness of the importance of OHM in diabetes management “Any medication you hate it … you cannot benefit from it.. Right? But if you decide to accept it and focus on your inner psychological acceptance everything will be fine” MASA 007, M, 66 years, MMAS 8, HbA1c 7.8%. Interviewees adhere to OHM in the anticipation of better results “Yes, I told you that I do not take the medications for the sake of today; I take them for the sake of the future.” MASA 182, M, 41 years, MMAS 6.75, HbA1c 7.2% Long-term use of OHM was associated with an optimistic belief of eventually being off the medication “I wish I could reach the point where I can control it with diet only… no medication” MASA 402, F, 58 years, MMAS 8, HbA1c 7%.	
Emotion	Positive emotions “I used to feel that my body is very heavy, buy when I monitored my diabetes and take my pills I felt more active, lively, and youthful…. You live happily, you feel like you can do anything, you sleep happily, and you’re happy with your family. MASA 402, F, 58 years, MMAS 8, HbA1c 7%.	Worry “Maybe I’m very careful. I want to decrease anxiety; I do not want to leave my medicine, but I do not want to focus too much, and I wonder if I’ve taken the pill. It’s my nature and it’s reflected on my medications.” MASA 168, F, 44 years, MMAS 6, HbA1c 8.8%. Frustration “As I’m a teacher, I have to wake up and eat my breakfast in the early morning. Some days I do not have an appetite but I have to force myself to eat in order to take my diabetic pills, which is really frustrating… and again at the dinner time I suffered also, because most of the times I force myself to eat just to take the medication.. I wish I was like normal people where I eat when I feel to and want to …” MASA 168, F, 44 years, MMAS 6, HbA1c 8.8%. Boredom “Honestly, I am bored of medications. I would like something that I could take once and that is it.” MASA 78, M, 26 years, MMAS 2.5, HbA1c 7%.
Beliefs about capabilities	OHM adherence as an easy behaviour to achieve. “To cut it short, there is no hardship in taking medicine.” MASA 169, M, 47 years, MMAS 8, HbA1c 7%. Self-confidence helped with OHM adherence. “Deep inside me I’m confident of myself and I know that it’s dangerous for me to stop it [OHM]. It’s not only dangerous; the pain will increase and everything else will increase.” MASA 65, F, 64 years, MMAS 8, HbA1c 8.2%.	
10. Reinforcement	Self-reward or taking one day off per week to eat sweets. “I always think of giving gifts to myself if I take my medication and controlled my sugar, like a grilled fish in the highest quality restaurant, or buying a new shemagh [Saudi men head cover] or besht [Saudi men clothing] just like I’m giving a gift to my wife or children…” MASA 166, M, 55 years, MMAS 8, HbA1c 7.5%. Encouraging letters or certificates from the UDC to the patients. “Why do not give a certificate from the UDC to my family… just like we do at the school, when I gave certificate to the student’s mother thanking her daughter performance, the daughter improves even more…it will cost nothing but I think it will support us” MASA 186,F, 41 years, MMAS 2.5, HbA1c 7.5. Monthly appointments with a dietician and close contact and support from the diabetic clinic. “There are moral [He means not material] incentives. I used to go to a dietician, and the appointment was every two weeks. I used to consider myself a challenge to the doctor, a case that didn’t lose weight sometimes. Sometimes she would get angry with me and that give me push to work harder.” MASA 110, M, 33 years, MMAS 3.75, HbA1c 7.7%.	
Skills	Knowing when and how to take their OHM. “[the question was how easy do you find taking diabetic medication] …very easy thanks to Allah [God]” MASA 006, M, 44 years, MMAS 7, HbA1c 7.4%. Practicing stress management techniques would help facing stress. “When I take the medication, and I remember that I took blood pressure medication also, I know that I do not want the pressure to rise, or the diabetes to rise too. I’m trying to avoid problems as much as I can.” MASA 125, M, 61 years, MMAS 7, HbA1c 8.4%.	
Social/professional role and identity	Interviewees who considered themselves as organized and committed to their plans, are motivated to review their medication and think about it like any other of their activities. "Why I'm taking my medication….? I do not have specific reasons..I was always been a person who like organization, and arranging stuff in their places.. even who visit me at my office they do not find even a piece of paper on my desk!..even my car everybody see it as very neat and organized" MASA 006, M, 44 years, MMAS 7, HbA1c 7.4%.	
Intentions	An intention to cure from diabetes “My goal, God willing, is to recover [from diabetes] and stop the medications.” MASA 007, M, 66 years, MMAS 8, HbA1c 7.8%. To attain and/or preserve good quality of life and avoid severe consequences to their health. “In all of our programmes, we set a general aim and a specific aim. The general aim is to preserve health; diabetes medications will be with you all your life. The specific aim is that you can live each stage with its good [he means good things in life], and this does not happen unless you take your diabetes medications and follow the doctor’s instruction.” MASA 006, M, 44 years, MMAS 7, HbA1c 7.4%.	

In the domain *behavioural regulation* it emerged that scheduling medication intake and the ability to develop a habitual behaviour were facilitators for OHA adherence.

*“In the first 1 to 3 years it’s a big deal*, *but after 15 years it became a habit*, *just like smoking; if I eat and don’t take it I think*, *“Where is the medication*? *Bring me the medication”* ID 125, M, 61 years MMAS 7, HbA1c 8.4%.

Unexpectedly, blood glucose monitoring and managing hypoglycemic were reported to be the barriers to OHA adherence.

*“I have my own device and the hospital gave me a device and chips so I kept checking the diabetes*. *I noticed that everything is fine*, *and that is it [the reason to discontinue the OHM]”* ID 143, M, 37 years, MMAS 3.5, HbA1c 6.9%.

When examining the factors related to the *social influence domain*, acting as a role model, the effect of family support, sharing medication at social events (with companions who had forgotten their OHAs) and having a supportive physician, were the main facilitators associated with OHA adherence.

*“At gatherings I say bring me my medicine to take away shame from the others*…*if those who hold college degree and also say bring me my medicine…”* ID 166, M, 55 years, MMAS 8, HbA1c 7.5%.

“*I once attended an event and the host [a friend] prepared a plate full of Glucophage [OHM] and said if anyone forgot to take it*, *here it is on the plate*, *out of hospitality*.*”* ID 019, M, 49 years, MMAS 8, HbA1c 8%.

Conversely, family gathering and holidays had detrimental effects on adherence.

*“I have to attend family gatherings*, *which creates pressure on me*. *I don’t like when people start asking me what medication I’m taking or what’s wrong with me*.*”* ID 186, F, 41 years, MMAS 2.5, HbA1c 7.5%

Furthermore, having a poor patient-physician relationship was perceived to have a determined effect on OHA adherence.

*“…*, *the first doctor wasn’t good*.. *you tell him that you feel dizzy*, *and he says don’t stop the medication*. *I didn’t go to him again and I keep monitoring myself*. *I decided that I will never go again…*..*”*. ID 78, M, 26 years, MMAS 2.5, HbA1c 7%.

*Knowledge* about diabetes and its management with OHAs influenced adherence and was often related to access to information. For example, family members’ lack of knowledge about the disease negatively impacted adherence, as did interviewee lack of information regarding OHAs, adherence and relevant lifestyle behaviour. Furthermore, knowledge gained from observing a family member's suffering from diabetic complications because of not adhering to the diabetic medication facilitated this participant to adhere to his medication.

*“Why should I commit [to take OHM]*, *because I lived through a tragedy*. *…*. *He [his father] got diabetic gangrene which required that one of his limbs be cut off*. *This horrified us*, *and later I discovered by chance that I have diabetes”* ID 019, M, 49 years, MMAS 8, HbA1c 8%.

The *environmental context and resources domain* includes the availability of material and/or social resources, to facilitate medication adherence. Factors identified were having trust in the centre and appropriate follow-up. Access to OHAs in the community pharmacies without prescription was considered to be a facilitator for OHA adherence.

*“… I take enough depending on the period I am travelling*. *If it runs out I get it from the pharmacy*, *as I already know the name of the medicine*. .*”* ID 125, M, 61 years, MMAS 7, HbA1c 8.4%.

The environmental context and resources domain also included the effect of religious beliefs on OHM adherence by increasing acceptance of the condition as well as of the medical recommendations by following the physician's instructions and maintaining the patients’ own health.

*“I take my medicine because it’s a way to worship Allah*, *because he says don’t throw yourselves in Tahluka [anything that would lead to your death]*.*”* ID 166, M, 55 years, MMAS 8, HbA1c 7.5%.

Conversely, psychosocial stress, witnessing accidents related to hypoglycemic events and prescriptions refill barriers such as restricted date and or duration of refilling a prescription, were identified as barriers to OHA adherence rel;ated to the environmental context and resources domain.

Furthermore, having to manage the multiple intake of several medications was identified as a barrier to OHA adherence related to the environmental context and resource domain.

*“I would like to take one pill instead of two; it has the same medical effect but it makes me feel better”*ID 110, M, 33 years, MMAS 3.75, HbA1c 7.7%.

*Beliefs about consequences* included consequences of having diabetes as well as consequences of using OHAs. Improved quality of life and the avoidance of adverse health outcomes and the need to use insulin, were factors which encouraged adherent behaviour.

*“It was a turning point in my life when the doctor said that if the pills don’t work we will have to initiate injections… it made me think I don’t want to take injections for the rest of my life*. *You can punish me anyway but don’t give me injections*.*”* ID 402, F, 58 years, MMAS 8, HbA1c 7%.

*“No*, *Alhamdulillah (thank God) I became better; I felt some muscle ache but now it is gone since I started taking the diabetes pills”* ID 184, F, 64 years, MMAS 7, HbA1c 8.7%.

However, fear of OHA side effects as well as being asymptomatic reduced adherence as the following quotation illustrate.

*“I didn’t take it [the OHM] for six months and didn’t feel anything*, *and when I took it I didn’t feel anything*. *When I took it I never felt anything*, *and even when I didn’t take it I felt the same”* ID 196, M, 63 years, MMAS 3, HbA1c 13.3%.

The *memory*, *attention and decision process domain* was clearly facilitated by each participants’ personal decision to adhere to OHAs to improve his/her quality of life. On the other hand, barriers to OHA adherence related to this domain were forgetfulness which interviewees experienced because they were tired and their decision to OHA due to stress and hypoglycemic side effects.

*“What can I do*? *I overslept*. *I don’t want to have dinner and I forgot my dose*. *This happens to me once or twice a month*.*”* ID 168, F, 44 years, MMAS 6, HbA1c 8.8%.

*”… I suffer from diabetes and obesity*, *and these medications contradict what I do*, *so they did not do me any good and I stopped taking them*.*”* ID 125, M, 61 years, MMAS 7, HbA1c 8.4%.

## Discussion

Despite free medical services and medication in Saudi Arabia, good OHA adherence among the studied cohort was low, i.e. 40%. This rate is lower than rates reported in India and Malaysia [11–13] but considerably higher than the 9% reported in the United Arab Emirates (UAE) [14] (Table 4). The UAE cohort was recruited from primary health care centres rather than a tertiary centre as in the current study, which may also have contributed to the difference in prevalence. Whilst several studies have assessed OHA adherence in Saudi Arabia, none focused on non-insulin users only [15–18]. Studies of Type 2 diabetic patients showed different behaviour toward medication-taking than patients who were using insulin plus OHA, with different adherence being associated with education and instruction provided to insulin users, as well as fear of hypoglycaemia and injections [19]. This study synthesized high-quality evidence about the level of OHA adherence in Type 2 diabetics who are not using insulin and identified factors affecting OHA adherence in the Saudi population.

**Table 4:** Cross-country comparison of prevalence of medication adherence using MMAS-8.

Study	Country	N	Morisky Medication Adherence Scale			
				Low MMAS<6 (%)	Moderate MMAS ≥6-<8 (%)	High MMAS = 8 (%)
Al-Qazaz 2011 [12]	Malaysia	540	Median (IQR) 6.5 (4.7–7.75)	NA	NA	NA
Arulmozhi 2014 [11]	India	150	Mean (SD) 6.6(2.0)	26.0	24.7	49.3
Manan 2014 [13]	Malaysia	179	Median (IQR) 7.8 (6.5–8.0)	52.0		48.0
Al-Haj Mohd 2016 [14]	United Arab Emirates	446	NA	64.57	26.46	8.97
Aloudah NM (Current study)	Saudi Arabia	395	Median (IQR) 7.0 (6.0–8.0)	23.3	36.7	40.0
				23.3	76.7	
				60.0		40.0

IQR; interquartile range, MMAS, Morisky Medication Adherence Score, NA; not applicable, SD; standard deviation.

In terms of demographic and clinical factors the current study demonstrated older age to be associated with better OHA adherence and this has been identified previously [11,14]. Low HbA1c was associated with better OHA adherence. The results of a recent systematic review showed a significant negative correlation between OHA adherence and HbA1c irrespective of the measure used [20]. However, it is to be noted that factors other than the level of OHA adherence, such as following a healthy lifestyle might result in better HbA1c levels [21]. Polypharmacy has previously been shown to be inversely associated with medication adherence [2] and this was also reflected in the current study both for total number of OHAs used as well as total number of medications (for any condition). Higher OHA adherence was associated with using fewer OHAs and fewer medications in general. In the current study, this association was not statistically significant for OHAs, possibly due to few multiple OHAs being used (Table 1). Gender, education, marital status, income, BMI, diabetes duration, completion of diabetes education program and enrollment in the home blood glucose monitoring were not statistically significant predictors of OHA adherence in the current study and reflects previous research which failed to identify any consistent association between OHA adherence and sociodemographic and disease factors [22].

Using a theory or theories in qualitative research design when attempting to increase knowledge on a specific area is a form of saving time and effort by considering a set of key variables that have emerged as relevant for the explanation of the behaviour [8]. The results of the MASA interview study derived from the TDF provide further depth and understanding to those of the cross-sectional study, in terms of factors influencing OHA adherence. Several studies conducted on Saudi participants have used qualitative measures to explore medication taking behaviour but either did not used theoretical underpinning [23] or used only one theory such as health belief model [24] or Modified Social Learning Theory [25].

Explaining behaviour and improving it through theory-based research has been used previously in medication adherence studies [26,27] but with limited benefit in changing targeted behaviour, which might be due to the large pool of theories or a limited understanding of which theory to choose and how to apply it to the problem studied [8]. The MASA Interview Study used the TDF to explore OHA adherence. The use of the TDF that integrates 33 psychological theories to understand behaviour was applicable and useful [8]. The TDF identified additional factors which illustrated that OHA adherence is a result of different interactions between patient and other factors, which might explain why most of the interventions targeting medication adherence have not been successful. The following section discusses some of these factors.

The interviewees expressed a need for building the habit of OHA adherence and this reflects studies with Canadians [28] and Turkish migrants [29], which demonstrated the importance of daily routine as well as being able to modify medication use in accordance with food intake.

Similar to findings of Mayberry et al [30], the interviewees in the current study reported positive family support as one of the important facilitators for OHA adherence. The concept of involving a role model in interventions that target behaviour change has been effective in changing physical activity and nutrition behaviours [31]. However, this approach has not been applied to medication adherence. Nevertheless, some of those interviewed during the current study believed that acting as a role model inspired other patients with diabetes to adhere to their medications. Using behaviour change techniques such as modeling using a role model might facilitate OHA adherence and could be explored further.

The patient-physician relationship is another important social factor raised by the interviewees, wherein a good relationship was reported as a facilitator and vice versa. Good communication between patient and physician increases patient’s confidence as well as their ability to cope with a chronic illness such as diabetes, thereby improving their medication adherence [32]. Therefore, appropriate training is needed for health care professionals supporting them to train and use skills to build rapport and engage in patient-centered care with their patients.

Gaps in knowledge about diabetes and OHAs were among the barriers identified as affecting adherence in this study; the association between appropriate knowledge and good medication adherence has been shown elsewhere [33]. Over half of our cohort were enrolled in the 5-day specialised education program, offered by the University Diabetes Centre responsible for their care, yet the interviewees identified knowledge gaps. The "one size fits all" approach might not be as effective as intended and specific intervention strategies that address individual patients’ needs should be considered. Additionally, pharmacist-led interventions have improved patients mediation adherence with diabetes [34] and could be considered as an adjunct to the support currently offered by the University Diabetes Centre.

Various beliefs about the consequences of diabetes and trying to avoid them were facilitators of OHA adherence. Guenette et al conducted six focus groups with 45 participants with Type 2 diabetes who had been taking OHM for more than three months and demonstrated that patients’ willingness to avoid long term complications was the most important factor associated with high OHA adherence [28]. Conversely, anticipated side effects are an important barrier to OHA adherence and have been reported among a wide range of populations [35–37]. The positive aspect of OHA adherence in improving quality of life should be addressed, particularly as medication adherence has been associated with enhanced quality of life among patients with diabetes [38].

Participants' religious beliefs seemed to influence their OHA adherence. Religious empowerment of medication adherence would encourage patients to schedule their diabetes management behaviours e.g. taking medications when going to the mosque, church, and temple, similar to what has been earlier reported by Hatahet al. from Malaysia [39].

Non-adherence to diabetes medication could either be intentional (decision-making) or un-intentional (forgetfulness) [30]. The current study reported forgetfulness as one of the unintentional OHA adherence barriers, whilst on the other hand, some interviewees intentionally decided not to adhere to their medication when experiencing side effects or during stressful events. Intentional non-adherence might explain the mismatch between medication adherence (MMAS-8) and diabetic control (HbA1c) of some interviewees, where some low adherent individuals (using MMAS-8) had good diabetic control (using HbA1c). However, as per the findings of Stack et al [40], unintentional nonadherence to OHAs was high in comparison with antihypertensive medications and statins. Therefore, intentional and unintentional adherence behaviours should be clearly differentiated among patients with diabetes since each requires different interventional approaches.

### Strengths and limitations

The cross-sectional study did not explore intentional versus un-intentional adherence. Reanalysis of the MMAS-8 to identify these percentages could be undertaken. The MASA interviews examined the patient perspectives regarding OHA adherence without differentiating between individuals with low and high adherence. Further analysis could be undertaken to compare the beliefs of low and high adherence.

MMAS-8 is a measure of self-reported behaviour and as such, outcomes could be biased. Social desirability bias and giving answers that favoured the behaviour of adherence and over estimation might have occurred in studies using self-reported measures [41]. However, this tool has been proven to have good reliability and validity [4]. This is a single centre study, conducted in a tertiary centre, as such, the results might not be generalisable to other patients with Type 2 diabetes who are cared for by non-tertiary or specialist centres. It is worthy of note, however, that despite being treated at a tertiary centre and having access to the educational programme, only 40% of patients in our study achieved high adherence rates.

## Conclusion

Low OHA adherence may contribute to the rates of morbidity and mortality observed amongst patients with Type 2 diabetes in Saudi Arabia. Low adherence is not solely an individual or patient-related problem. Physician, societal and organizational factors influence this behaviour, therefore a comprehensive, multi-faceted approach is required to optimise the benefits of OHA medication.

## Supporting information

S1 Table Study interview topic guide. (DOCX)

S2 Table Interviewees' demographic and diabetic characteristics. (DOCX)

S3 Table COREQ (COnsolidated criteria for REporting Qualitative research) checklist. (DOCX)
