# Supplementary material for: Medication adherence among patients with Type 2 diabetes: A mixed methods study
Source: PLoS One. 2018 Dec 11;13(12):e0207583. doi: 10.1371/journal.pone.0207583 (PMC6289442; doi:10.1371/journal.pone.0207583)
Supplement: S1 Table — (DOCX) [file pone.0207583.s001.docx]

**Supporting information 1** Study interview guide *NB: over 2 pages*

Tell me the reasons why you need your diabetic medication?

How do you know how to take your diabetic medication? (prompt: how, when, in relation to food, daily activities)

How easy do you find taking diabetic medication? (Prompt: How difficult)

How does this medication feel to you?

Is it in line with your values?

Do you feel that it is an appropriate part of your life to take this medication?

What problems have you encountered while taking your medication?

What would help you in taking your medication as prescribed?

How capable are you continuing to take your medication?

How well capable do you feel about taking your diabetic medication?

How confident are you about taking your medication despite the difficulties encountered?

What do you think will happen if you take your medication as prescribed?(prompts: re themselves, family, health-care system, short and long term consequences)

What do you think will happen if you do not take your medication as prescribed? (cost and consequences)

Do advantages of taking your medication compensate for the disadvantages of it?

When you do not take your medication, how do you feel? (prompts: what do you feel if you take your medication)

Are there rewards associated with taking your medication?

Are there other things you want to do that might be affected with taking your medication?

How much do you want to take your medication?

How much do you like to take your medication?

What makes you take your medication? (prompts: feel better, control, increase life expectancy)

Is taking your medication something you usually do?

When, where? How do you take your medication?

How much effort do you spend taking your diabetic medication as prescribed? (prompt: actively think about it)

How do you remember to take your medication?

What makes you decide not to take your medication? (Prompt: competing tasks/time, side effects)

Could you think about an occasion you did not take your medication?

Did you consciously decide not to take your medication?

Do you think you have what is needed to take your diabetic medication as prescribed?

Are there competing tasks (constraints) that prevent you from taking your medication?

Do you feel you have the necessary support/help available to take your diabetic medication as prescribed?

What do others think about you taking your medication (husband, kids, GPs,...ect)

To what extent do social influences, e.g. People around you, facilitate or hinder taking your medication? (Prompts: family, friends, other patients....)

Will you follow others taking their diabetic medication as prescribed? (Have a role model)?

Dose taking your medication affect your emotions? How? (prompt: )

To what extent do emotional factors facilitate or hinder taking your medication?

What actions are needed to achieve the goal of taking medication?

Are there procedures/things you do that encourage/facilitate medication taking?

Is this a new behaviour (taking diabetic medication as prescribed) or an existing behaviour that needs to become a habit?

How long are changes to take?

Can you suggest systems for maintaining long term changes for medication taking as a habit?

What is it about taking this medication that makes it more easy/difficult? (changing the environment)
