# Supplementary material for: Medication adherence among patients with Type 2 diabetes: A mixed methods study
Source: PLoS One. 2018 Dec 11;13(12):e0207583. doi: 10.1371/journal.pone.0207583 (PMC6289442; doi:10.1371/journal.pone.0207583)
Supplement: S2 Table — (DOCX) [file pone.0207583.s002.docx]

| **ID** | **Age years** | **Gender** | **BMI kg/m^2^** | **Education** | **Marital status** | **Income Category† SR** | **Duration of diabetes years** | **OHM N** | **OHM duration years** | **Other meds N** | **Five**  **-day education** | **HBGM** | **HbA1c %‡** | **MMAS-8 (0-8)** **§** |
| --- | --- | --- | --- | --- | --- | --- | --- | --- | --- | --- | --- | --- | --- | --- |
| 6 | 44 | M | 44.5 | ≥College | Married | 3 | 11 | 2 | 11 | 4 | No | Yes | 7.4 | 7 |
| 7 | 66 | M | 39.7 | ≥College | Married | 2 | 17 | 2 | 17 | 8 | Yes | Yes | 7.8 | 8 |
| 19 | 49 | M | 37.2 | ≥College | Married | 1 | 5 | 2 | 5 | 1 | No | No | 8 | 8 |
| 64 | 57 | F | 37.6 | ≥College | Married | 3 | 7 | 2 | 7 | 5 | Yes | Yes | 8.1 | 6.75 |
| 65 | 64 | F | 26.4 | ≤High School | Widow | 1 | 16 | 1 | 16 | 3 | Yes | Yes | 8.2 | 8 |
| 78 | 26 | M | 30.5 | ≤High School | Unmarried | 3 | 7 | 1 | 5 | 0 | No | No | 7 | 2.5 |
| 110 | 33 | M | 32.4 | ≥College | Divorced | 4 | 5 | 3 | 3 | 0 | Yes | Yes | 7.7 | 3.75 |
| 125 | 61 | M | 30.9 | ≤High School | Married | 3 | 15 | 3 | 15 | 2 | Yes | No | 8.4 | 7 |
| 143 | 37 | M | 30.2 | ≤High School | Married | 2 | 14 | 1 | 14 | 2 | No | No | 6.9 | 3.5 |
| 166 | 55 | M | 31.7 | ≥College | Married | 4 | 18 | 2 | 15 | 2 | Yes | Yes | 7.5 | 8 |
| 168 | 44 | F | 26.2 | ≥College | Divorced | 3 | 15 | 2 | 15 | 2 | No | Yes | 8.8 | 6 |
| 169 | 47 | M | 28.4 | ≤High School | Married | 2 | 6 | 1 | 1.5 | 4 | No | No | 7 | 8 |
| 182 | 41 | M | 34.5 | ≥College | Married | 3 | 6 | 1 | 5 | 2 | Yes | No | 7.2 | 6.75 |
| 184 | 64 | F | 42.3 | None | Married | 2 | 4 | 3 | 4 | 1 | Yes | Yes | 8.7 | 7 |
| 186 | 41 | F | 26.7 | ≥College | Married | 4 | 3 | 1 | 3 | 4 | Yes | Yes | 7.5 | 2.5 |
| 196 | 63 | M | 20.3 | ≤High School | Unmarried | 3 | 20 | 3 | 20 | 4 | Yes | No | 13.3 | 3 |
| 402 | 58 | F | 23.7 | ≥College | Married | 5 | 20 | 1 | 5 | 2 | Yes | No | 7 | 8 |
| 437 | 57 | F | 31.6 | ≤High School | Widow | 1 | 18 | 1 | 18 | 8 | No | No | 7 | 8 |
| pilot 1 | 48 | F | NA | ≥College | Married | 3 | 14 | 2 | 16 | 0 | NA | NA | NA | 8 |
| pilot 2 | 72 | F | NA | ≤High School | Married | 4 | 12 | 1 | 12 | 5 | NA | NA | NA | 8 |

**Appendix 2** Interviewees' demographic and diabetic characteristics

†Income (1 is ≤5000, 2 is >5000-10000, 3 is >10000-20000, 4 is >20000-35000, 5 is >35000)

HbA1c, glycosylated haemoglobin; HBGM, home blood glucose monitoring; MMAS, Morisky Medication Adherence Scale; MRCI, Medication Regimen Complexity Index (higher is more complex); NA, not applicable, N, number; OHM, oral hypoglycaemic medication; SR, Saudi riyals. ‡Goal of therapy in UDC >7%.§MMAS=8 high adherence, MMAS 6 to <8 is moderate adherence, <6 is low adherence.
